# A limited sampling strategy for estimation of the area under the curve (0 to 8 hours) of mycophenolic acid administered three times daily to liver transplant recipients

**DOI:** 10.3109/03009734.2010.523801

**Published:** 2011-02-11

**Authors:** Bernardino Marcos, Lorena Bouzas, J. Carlos Tutor

**Affiliations:** Unidad Monitorización Fármacos, Laboratorio Central, Hospital Clínico Universitario, Instituto de Investigación Sanitaria (IDIS), Santiago de CompostelaSpain

**Keywords:** Area under the curve, dosing frequency increase, drug exposure, gastrointestinal side-effects, mycophenolic acid

## Abstract

**Objectives:**

Gastrointestinal side-effects caused by mycophenolic acid (MPA) are frequent in liver transplant recipients, and in these cases a switch from two to three daily doses is usually recommended. However, a limited sampling strategy for the estimation of MPA area under the curve from 0 to 8 hours (AUC_(0–8h)_) has not been made.

**Design and methods:**

In 22 liver transplant patients who were administered MPA three times daily, the trapezoidal extrapolated MPA AUC_(0–8h)_ values using a sampling time from 0 to 2 hours were calculated.

**Results:**

A tentative therapeutic range for MPA AUC_(0–8h)_ of about 20–40 μg.h/mL is proposed, and in the 13 patients with supratherapeutic values the total leukocyte blood count was significantly lower than in the 9 patients with AUC_(0–8h)_ ≤ 40 μg.h/mL (*P* < 0.001). Significant negative correlations were found between the total leukocyte blood count and the MPA trough levels (*r* = −0.458; *P* < 0.05), AUC_(0–8h)_ (*r* = −0.479; *P* < 0.05), and AUC_(0–2h)_ (*r* = −0.437; *P* < 0.05). A significant correlation was found between the trapezoidal extrapolated AUC_(0–8h)_ and trapezoidal AUC_(0–2h)_ results (*r* = 0.850; *P* < 0.001).

**Conclusions:**

The trapezoidal extrapolated AUC_(0–8h)_, and possibly trapezoidal AUC_(0–2h)_, may be useful for routine therapeutic MPA monitoring in liver transplant recipients in which the dosing frequency is increased from twice to three times a day.

## Introduction

Mycophenolic acid (MPA) is the active constituent of mycophenolate mofetil (MMF), which is a common component of the immunosuppressive regimens following transplantation. With the increasing use of MPA, the need for more accurate drug dosage has become evident, and the current scientific evidence for its concentration-controlled dosing in solid organ transplantation has been recently reviewed (1). MPA is usually administered in two daily doses, and the full MPA area-under-the-curve (AUC) from 0 to 12 hours (AUC_(0–12h)_) is considered the best measure of overall drug exposure; however, it is an impractical monitoring strategy for everyday clinical use (1). Limited sampling from the first few hours post-dose makes it possible to predict the full MPA AUC, and the most promising results to date regarding therapeutic MPA monitoring come from these limited sampling strategies (1,2). The majority of proposed limited-sampling strategies for estimating MPA AUC_(0–12h)_ was developed for kidney transplant recipients, and few algorithms for liver transplant patients are included by Bruchet and Emson in their recent systematic review (3).

The side-effects of MPA mainly involve the gastrointestinal tract (diarrhoea, nausea, abdominal discomfort) and bone-marrow (leucopenia, thrombocytopenia), but do not normally require the immunosuppressive agent to be changed (1,4). Diarrhoea and vomiting have a high prevalence among liver transplant patients receiving MMF orally (1,5), and these side-effects usually respond to dose reduction or switching from two to three divided daily doses (5). However, at least to our knowledge, a simplified sampling time profile for the estimation of MPA AUC from 0 to 8 hours (AUC_(0–8h)_) in liver transplant recipients treated with MMF three times daily has not been previously reported.

The routine application of abbreviated-sampling time strategies to estimate MPA AUC is feasible, if all samples can be taken within a 2-hour window (6). The aim of our study was to establish a trapezoidal extrapolated AUC_(0–8h)_ using a sampling time of 0 to 2 hours for the therapeutic MPA monitoring in liver transplant recipients, who were administered MPA every 8 hours due to gastrointestinal drug side-effects using the typical daily dose. The results of the trapezoidal extrapolated AUC_(0–8h)_ simplified strategy were compared with those of the trapezoidal AUC from 0 to 2 hours (AUC_(0–2h)_), which has been recently used for therapeutic drug monitoring of MPA administered in two daily doses to liver transplant patients (7).

## Patients and methods

MPA levels were monitored in 22 maintenance liver transplant recipients (17 males and 5 females) with a mean (± SEM) age of 58.1 ± 2.4 years, receiving daily oral treatment with MMF (CellCept, Hoffman La Roche, Basel, Switzerland) at an 8-hour interval in monotherapy (*n* = 6), or together with cyclosporin (*n* = 4) or tacrolimus (*n* = 12). In all of the cases, the reason for switching the MMF dosage from two to three divided daily doses was due to MPA side-effects involving the gastrointestinal tract (diarrhoea and nausea). After the MPA steady-state was achieved, blood samples were taken in BD Vacutainer tubes containing K_3_EDTA as anticoagulant, immediately before the next dose of MMF (C_0_), and half an hour (C_0.5_) and 2 hours (C_2_) post-dose. This study was carried out according to the good practice rules for investigations in humans of the Conselleria de Sanidade (Regional Ministry of Health) of the Xunta de Galicia, Spain.

MPA plasma concentrations were determined in duplicate using the EMIT 2000 Mycophenolic Acid Assay in a Dimension Xpand Plus analyzer (Siemens Healthcare Diagnostics Inc., Newark, DE, USA). In accordance with the procedure developed in kidney transplant recipients by Hale et al. (8), the AUC from 0 to 2 hours (AUC_(0–2h)_) was calculated using the linear trapezoidal rule, and the AUC values were extrapolated from 0 to 8 hours (AUC_(0–8h)_) considering the mathematically estimated concentrations at 6 (C_6_) and 8 (C_8_) hours. According to the characteristics of the MPA concentration-time profiles in liver transplant recipients (9), the C_6_ and C_8_ concentrations were calculated using the expressions: C_6_ = 1.25C_0_ + 0.15, and C_8_ = C_0_. The blood counts of total, polymorphonuclear (PMN) and mononuclear (MN) leukocytes, and platelets were carried out in an Advia 2120 Hematology System from Siemens Healthcare Diagnostics Inc.

Statistical analysis of the data was carried out using the Microsoft Excel (v. 5.0) package, and the Kolmogorov-Smirnov test was applied to check for normality. MPA levels and AUC data had Gaussian distributions, and consequently Pearson’s correlation coefficient, linear regression, and S_y.x_ as measure of dispersion, were used. In other cases the Spearman’s correlation coefficient was used. The results are expressed as mean ± SEM (median).

## Results

The generally considered therapeutic window for MPA AUC_(0–12h)_ is 30–60 μg.h/mL (10,11), and consequently, for the maintenance of an analogous daily drug exposure (cumulative 24 hours AUC), a tentative therapeutic interval for MPA AUC_(0–8h)_ of around 20–40 μg.h/mL may be proposed.

**Figure 1.**
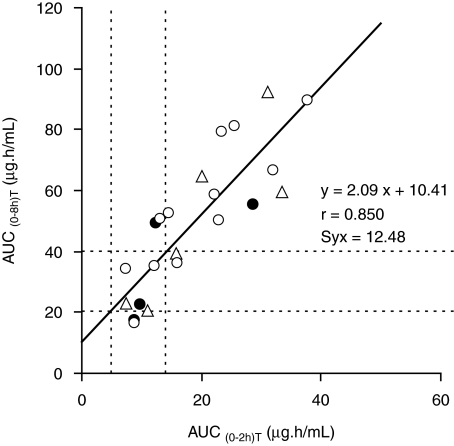
Correlation and regression between the trapezoidal mycophenolic acid (MPA) AUC_(0–2h)_ and trapezoidal extrapolated MPA AUC_(0–8h)_ in liver transplant recipients treated three times daily with mycophenolate mofetil (MMF) in monotherapy (Δ), or co-medicated with cyclosporin (•) or tacrolimus (○). The dashed lines correspond to the tentative therapeutic ranges for MPA AUC_(0–2h)_ and AUC_(0–8h)_.

The relationship between the trapezoidal extrapolated MPA AUC_(0–8h)_ and the trapezoidal AUC_(0–2h)_ values is shown in Figure 1, and, in accordance with the linear regression equation, the estimated therapeutic range for AUC_(0–2h)_ may be about 5–14 μg.h/mL. In the 22 patients studied the mean trapezoidal extrapolated MPA AUC_(0–8h)_ was 49.7 ± 4.9 μg.h/mL (range 16.5–92.2 μg.h/mL), and in 13 cases the values were supratherapeutic (>40 μg.h/mL). For the MPA trapezoidal AUC_(0–2h)_ a mean value of 18.9 ± 2.0 μg.h/mL (range 7.3–37.9 μg.h/mL) was obtained, and also in 13 cases the values were supratherapeutic (>14 μg.h/mL). However, a modest concordance was observed in the classification of AUC_(0–2h)_ and AUC_(0–8h)_ values as subtherapeutic, therapeutic or supratherapeutic (Figure 1).

Significant correlations were found between the trapezoidal extrapolated MPA AUC_(0–8h)_ and C_0_ (AUC_(0–8h)_ = 9.79C_0_ + 16.24; *r* = 0.909; *P* < 0.001; S_y.x_ = 9.9 μg.h/mL), C_0.5_ (AUC_(0–8h)_ = 1.77C_0.5_ + 27.38; *r* = 0.636; *P* < 0.01; S_y.x_ = 18.3 μg.h/mL), and C_2_ (AUC_(0–8h)_ = 4.42C_2_ + 18.20; *r* = 0.753; *P* < 0.001; S_y.x_ = 15.6 μg.h/mL). Similarly, significant correlations were also found between the trapezoidal MPA AUC_(0–2h)_ and C_0_ (AUC_(0–2h)_ = 3.03C_0_ + 8.50; *r* = 0.689; *P* < 0.001; S_y.x_ = 6.99 μg.h/mL), C_0.5_ (AUC_(0–2h)_ = 1.06C_0.5_ + 5.41; *r* = 0.939; *P* < 0.001; S_y.x_ = 3.31 μg.h/mL), and C_2_ (AUC_(0–2h)_ = 0.99C_2_ + 11.78; *r* = 0.415; *P* ≈ 0.05; S_y.x_ = 8.78 μg.h/mL).

With respect to the 13 cases with trapezoidal extrapolated MPA AUC_(0–8h)_ > 40 μg.h/mL, in the 9 cases with trapezoidal extrapolated MPA AUC_(0–8h)_ ≤ 40 μg.h/mL the MPA C_0_ level (1.7 ± 0.2 μg/mL (1.9 μg/mL) versus 4.9 ± 0.5 μg/mL (4.4 μg/mL)) was significantly lower (*P* < 0.001), and the total leukocyte blood count (7504 ± 687/μL (7600/μL) versus 5131 ± 438/μL (4930/μL)) was significantly higher (*P* < 0.05). However, for the blood count of PMN, and MN leukocytes, and platelets, the differences were not significant. Table I shows the correlation coefficients of the blood count of total, PMN, and MN leukocytes, and platelets with the MPA C_0_, C_0.5_, and C_2_ levels, and AUC_(0–8h)_ and AUC_(0–2h)_.

**Table I.** Correlation coefficients of the blood count of total, polymorphonuclear (PMN), and mononuclear (MN) leukocytes, and platelets with mycophenolic acid levels and AUC_(0–2h)_ and AUC_(0–8h)_ (*n* = 22).

	Total leukocytes	PMN	MN	Platelets
C_0h_	−0.458 (*P* = 0.032)	−0.399 (*P* = 0.066)	−0.449 (*P* = 0.036)	0.197 (*P* = 0.379)
C_0.5h_	−0.364 (*P* = 0.096)	−0.201 (*P* = 0.371)	−0.328 (*P* = 0.136)	0.351 (*P* = 0.109)
C_2h_	−0.312 (*P* = 0.157)	−0.374 (*P* = 0.086)	0.028 (*P* = 0.903)	0.020 (*P* = 0.931)
AUC_(0–2h)_	−0.437 (*P* = 0.042)	−0.324 (*P* = 0.141)	−0.295 (*P* = 0.183)	0.238 (*P* = 0.286)
AUC_(0–8h)_	−0.479 (*P* = 0.024)	−0.419 (*P* = 0.052)	−0.299 (*P* = 0.176)	0.205 (*P* = 0.360)

## Discussion

It has been suggested that concentrations of MPA in the immediate post-dose period may not accurately correlate to total drug exposure, because these concentration–time points do not capture a second peak concentration due to the drug reabsorption into circulation after its biliary excretion (2). However, substantial research has been conducted to develop limited-sampling strategies to estimate MPA AUC in kidney, heart, liver, and lung transplantation, and it seems that concentrations in the immediate post-dose period can accurately predict total MPA AUC_(0–12h)_ (3).

The clinical usefulness of the MMF original dosing protocol change from two to three times daily, overcoming the disadvantage of short MPA half-life and improving immunosuppression in haematopoietic cell transplantation, has been the subject of discussion (12–14). With respect to solid organ transplant recipients, treating physicians usually increase the dosing frequency to three times a day if MPA gastrointestinal side-effects are present (5). In these clinical conditions, it may be important to have a simplified strategy available for the MPA AUC_(0–8h)_ estimation applicable to the routine drug exposure monitoring.

At present, the selection of appropriate target ranges for MPA AUC_(0–12h)_ is challenging, as the range of 30–60 μg.h/mL was based on the use of MMF in the early post-renal transplant period with concomitant cyclosporin (3). Similarly, the analytical method used for the MPA determination should be considered, as the results produced by the enzyme multiplied enzyme immunoassay (EMIT) are higher than those of the chromatographic-based techniques, because the antibody used in the immunoassay has cross-reactivity with the pharmacologically active MPA acyl glucuronide metabolite (1,3). In any case, we considered the therapeutic range of 30–60 μg.h/mL for MPA AUC_(0–12h)_ due to its wide acceptance (7,10–12,15,16). Consequently, for the maintenance of an analogous daily drug exposure, a therapeutic range of 20–40 μg.h/mL may be proposed for the MPA AUC_(0–8h)_. The increase of the MMF dosing frequency could statistically maintain higher MPA trough levels and steady-state concentrations (AUC/dosing interval) (13), and in 13 cases (59%) the trapezoidal extrapolated MPA AUC_(0–8h)_ values were supratherapeutic, suggesting the suitability of a drug dosage adjustment.

A modest concordance was found between subtherapeutic, therapeutic, and supratherapeutic AUC_(0–8h)_ and AUC_(0–2h)_ values (Figure 1). Co-administration of other immunosuppressant agents may influence MPA exposure, as is clearly shown for cyclosporin with 30%–40% lower dose-normalized MPA exposure (1). However, in the group of patients studied, concomitant cyclosporin administration does not appear to modify significantly the relationship between the MPA AUC_(0–2h)_ and AUC_(0–8h)_ (Figure 1).

Therapeutic monitoring of MPA in liver transplantation has predominantly used the trough concentration (C_0_), although additional AUC data exist in some studies, suggesting an acceptable correlation of C_0_ with AUC_(0–12h)_ (1) and AUC_(0–2h)_ (7). In our study, the higher correlation coefficients were found between MPA C_0_ and AUC_(0–8h)_ (*r* = 0.909; *P* < 0.001), and C_0.5_ and AUC_(0–2h)_ (*r* = 0.939; *P* < 0.001).

It has been reported that in liver transplant recipients treated with two daily doses of MMF, the MPA pharmacokinetic parameters, C_0_, C_max_, and AUC_(0–12h)_, were significantly higher in patients with side-effects than in those with an uneventful outcome (16). However, Shin et al. (7) recently described no significant relationships among MPA C_0_ levels or AUC_(0–2h)_ with the degree of leucopenia, diarrhoea, or infection. In our group of patients with MPA AUC_(0–8h)_ >40 μg.h/mL, the blood count of total leukocytes was significantly lower than in the group of patients with AUC_(0–8h)_ ≤40 μg.h/mL (*P* < 0.05). Similarly, significant negative correlations between the blood count of total leukocytes, with the MPA trough levels, AUC_(0–8h)_, and AUC_(0–2h)_ were found (Table I).

Regional concentrations of MPA in gastrointestinal epithelial cells, not reflecting systemic exposure, may contribute to undesirable gastrointestinal events (16). In accordance with Brunet et al. (17), gastrointestinal side-effects of MPA in liver transplant recipients were associated with high drug levels obtained 40 min after dose, but not with AUC_(0–12h)_ or C_0_. These results may explain the beneficial effect of the increase of MPA dosing frequency, maintaining an analogous daily drug exposure, on the diarrhoea, nausea, and vomiting. Trapezoidal extrapolated AUC_(0–8h)_ may be used for the routine MPA exposure monitoring in liver transplant recipients treated with this drug three times daily. Further studies on a larger number of transplant patients are necessary for the evaluation of the clinical usefulness of the trapezoidal MPA AUC_(0–2h)_ in comparison with the estimated AUC_(0–8h)_ or AUC_(0–12h)_ using limited sampling strategies.
